# Improving fermentation quality, aerobic stability, and microbial community stabilization of triticale by *Amomum villosum* essential oil

**DOI:** 10.1128/spectrum.01302-24

**Published:** 2025-05-21

**Authors:** Maoya Li, Yao Lei, Yulian Chen, Qiming Cheng, Xiaoqing Zhang, Chao Chen, Ping Li, Jiachuhan Wang, Hui Li, Yuanyuan Zhao, Xiangjiang He, Zhijun Wang, Wei Yan

**Affiliations:** 1 College of Animal Science, Guizhou University 655623, Guiyang, China; 2 Institute of Grassland Research of Chinese Academy of Agricultural Sciences, Hohhot, China; 3 College of Grassland, Resources and Environment, Key Laboratory of Forage Cultivation, Processing and High Efficient Utilization of Ministry of Agriculture, and Key Laboratory of Grassland Resources, Ministry of Education, Inner Mongolia Agricultural University 117454 , Hohhot, China; 4 Inner Mongolia Academy of Science and Technology, Hohhot, China; Consejo Superior de Investigaciones Cientificas, Villaviciosa, Asturias, Spain

**Keywords:** triticale silage, *Amomum villosum *essential oil, microbial community, aerobic stability, *Lentilactobacillus buchneri*, vanillic acid

## Abstract

This study aimed to examine the impacts of *Lentilactobacillus buchneri* (LB), *Amomum villosum* essential oil (AVEO), and vanillic acid (VA) on the fermentation traits, aerobic stability, and microbial community composition of ensiled triticale samples throughout the duration of air exposure. The chopped triticale was thoroughly blended, and the mixture was subjected to the following experimental treatments: CK, purified water at 1 mL kg^−1^ fresh weight (FW); AVEO, application at 1 mL kg^−1^ FW; LB, application at 1 × 10^6^ CFU/g FW; and VA, application at 1 mL kg^−1^ FW. During the aerobic exposure phase, the dry matter and starch contents of the additive groups were greater, and the neutral detergent fiber content was lower than that of the control group (*P* < 0.05), particularly in the AVEO treatment group. The pH value, lactic acid concentration, and aerobic stability were also greater (*P* < 0.05) in the AVEO treatment group than in the other treatment groups. Compared with the control silage, the additive groups presented greater *Bacillus* and *Lactiplantibacillus* abundances, whereas the additive groups presented lower *Sphingomonas* and *Stenotrophomonas* abundances. The AVEO-treated silage had greater *Lactiplantibacillus* and *Bacillus* abundances and lower *Sphingomonas* and *Stenotrophomonas* abundances. Compared with the control silage, the additive groups presented greater *Rhizopus* abundances, while the additive groups presented lower *Monascus* and *Cladosporium* abundances. The AVEO-treated silage had more *Rhizopus* and fewer *Monascus* and *Cladosporium* abundances. The use of AVEO in triticale silages resulted in increased aerobic stability, while also reducing the presence of harmful microbial communities.

**IMPORTANCE:**

The utilization of ensiling for the preservation of forage has been demonstrated to be an effective and environmentally sustainable approach. Nevertheless, ensiled samples will inevitably come into contact with air during use or under unfavorable circumstances, leading to aerobic deterioration. AVEO helped maintain a high relative abundance of *Lactiplantibacillus* and *Bacillus* after aerobic exposure, suggesting that AVEO acts as an enrichment agent for *Lactiplantibacillus* and *Bacillus* in triticale silage. The AVEO treatment group also had a greater relative abundance of *Rhizopus*, resulting in the continuous generation of LA under aerobic conditions. This led to the maintenance of an acidic environment, inhibition of fungal proliferation, and delay in aerobic deterioration. These findings demonstrate that AVEO has the ability to positively impact the microbial community of triticale silage and thus improve fermentation quality and aerobic stability. This study provides a scientific foundation for producing triticale silages of superior quality.

## INTRODUCTION

Silage is a crucial feed for ruminants such as cattle, sheep, and horses worldwide. It serves as a significant source of nutrients, energy, and fiber (1). In light of anticipated feed shortages and concerns about food security, the need for the production of superior silage will increase significantly in the coming years (2). Triticale cultivation has established a substantial international presence, encompassing more than 30 countries globally. With the need for agricultural structural adjustment and ecological construction in China, triticale is currently used mainly as forage in animal husbandry and is rotated or double-cropped with corn, cotton, etc., and the planting area is constantly expanding (3). The cultivated land area of triticale is approximately 3.7 million hectares, reaching an annual output of 12 million tons (3).

Triticale is a new species formed by the hybridization of *Triticum* and *Secale*, which combines the advantages of high nutritional value and high yield of *Triticum aestivum* with the strong stress resistance of *Secale cerale*. This species is an annual member of the *Poaceae* family and is categorized as cool-season forage grass. It effectively exploits the cool seasons of winter and spring (otherwise known as winter fallow periods) for forage production, furnishing high-quality green forage to livestock during the winter and spring dormant periods of native grasses (4). Additionally, as a green forage grass, it boasts of an early harvest period, facilitating staggered forage production. Triticale has noteworthy characteristics, including a relatively high production capacity, a substantial starch content, a significant presence of water-soluble carbohydrates (WSC), and powerful a-amylase activity (4). Thus, triticale is recognized as a crop that helps alleviate the scarcity of livestock feed (4, 5).

Because triticale harvesting occurs on a seasonal basis, it is important to prioritize suitable storage methods to maintain an uninterrupted supply of feed. The utilization of ensiling for the preservation of forage has been demonstrated to be an effective and environmentally sustainable approach (1–3). This ensiling process results in a low-pH environment that inhibits the development of unwanted bacteria while simultaneously increasing the breakdown of lignocellulose (6, 7). The addition of lactic acid bacteria (LAB) and enzymes to triticale for ensiling reduces the fiber content of triticale and prolong its storage time (3). Nevertheless, ensiled samples will inevitably come into contact with air during use or under unfavorable circumstances, leading to aerobic deterioration. Aerobic spoilage of silage causes aerobic microorganisms to become active, leading to their seizure of resources and space from LAB and increasing the consumption of nutrients in silage (8). The aerobic degradation of silage not only results in the depletion of nutrients but also hampers the efficiency of feed utilization and diminishes the production capacity of ruminant animals (9). Hence, it is necessary to increase the aerobic stability of triticale silage to minimize the possibility of secondary fermentation. The combination of *Bacillus coagulans* and *Lactiplantibacillus plantarum* may lead to improved fermentation quality and aerobic stability in ensiled triticale (4). However, no attention has yet been given to the effects of additives other than microbial inoculants on the aerobic deterioration of triticale silage, including the influence of the microbial structure.

To increase the aerobic stability of silage, a variety of additives have been used. The integration of heterofermentative LAB into the ensiling process is a technique that is commonly used for enhancing the aerobic stability of silage samples (10). Heterofermentative LAB employ hexoses as substrates to produce lactic acid (LA), acetic acid (AA), and ethanol. AA has been shown to increase aerobic stability, leading to a decrease in nutritional losses in silage after air exposure, as well as cellulose constituent degradation (11). *Lentilactobacillus buchneri* (LB) favors the inhibition of silage deterioration during the aerobic exposure phase (12). Vanillic acid (VA) is known for its characteristic creamy flavor (13). VA exhibits anti-inflammatory and antibacterial characteristics in addition to antioxidant and antihypertensive properties (14). According to previous reports, VA can improve the retention of nutrients in stylo silage and minimize the presence of harmful bacteria that might negatively impact fermentation quality (15). Specifically, VA can stimulate the production of laccase enzymes, which are beneficial for fiber degradation and have potential for use in various industrial sectors (16).

The use of aromatic plants and their derivatives as additives for ensiling fermentation has garnered significant interest owing to their observed antifungal, antibacterial, and antioxidant capabilities (17). In recent years, interest in essential oils within the livestock industry has increased, and essential oils are considered promising alternatives to antibiotics (17). Furthermore, studies have revealed that plant-based essential oils (EOs) can effectively act as modulators of ruminal fermentation, thereby increasing dry matter intake and improving the digestibility profiles of starch, fiber, and nitrogen in ruminants (18). By disrupting the permeability of cell membranes, EOs are able to control the mechanism that is responsible for the transfer of nutrients, and they effectively impede the growth and multiplication of bacteria that are responsible for the spoilage of silage (18, 19). Furthermore, it has been established in prior research that the incorporation of lemon EO into sugarcane silage results in enhanced aerobic stability and fermentation quality, diminished nutrient losses, and an accelerated rate of fiber degradation (20). *Amomum villosum Lour*., a fragrant medicinal herb commonly employed in traditional Chinese medicine, exhibits health-promoting properties such as heat-clearing and summer-heat-relieving effects, enhancing body metabolism, and alleviation of bloating (21). *Amomum villosum* essential oil (AVEO) contains flavonoids, phenolic compounds, terpenoids, etc., which have antibacterial, antifungal, antioxidant, and other biological activities (21). Multiple studies have shown that AVEO can improve the oxidative stability of sunflower oil because of its good antioxidant effects, and AVEO has the potential to replace synthetic antioxidants as a natural antioxidant-containing product (22). The use of AVEO as a silage additive has been shown to enhance the establishment of LAB but also impedes the proliferation of deleterious microorganisms in silage, according to the findings of our previous study (23).

In summary, although the application of AVEO and VA enhances the overall fermentation quality of silage, there have been no published studies on the aerobic stabilization of silage with AVEO and VA. This study aimed to investigate the impacts of LB, VA, and AVEO on the fermentation quality and aerobic stability of triticale silage, with a particular emphasis on the microbial community, lignocellulose degradation rate, and nutrient composition. We hypothesize that the application of LB, VA, and AVEO can improve the fermentation quality and increase the aerobic stability of triticale silage through modulation of the microbial community composition.

## MATERIALS AND METHODS

### Preparation of triticale samples for ensiling pretreatment

Triticale harvest took place on 21 June 2022, and the plants were harvested from three plots that were chosen at random. The harvest site experiences an annual mean temperature of approximately 15°C and an annual mean precipitation of 1,400 mm. The triticale was harvested at the maturity stage. The harvesting procedure consisted of leaving a stubble that was 10 centimeters long, after which the triticale was cut into filaments that were between 1 and 2 centimeters in length. The fresh triticale had a moisture content of 70.25% dry matter (DM), a crude protein (CP) content of 10.79% DM, a WSC content of 5.26% DM, a neutral detergent fiber (NDF) content of 69.92% DM, an acid detergent fiber (ADF) content of 46.07% DM, a hemicellulose content of 23.85% DM, and a starch content of 27.79% DM. The chopped triticale was thoroughly blended, and the mixture was subjected to the following experimental treatments: (i) control (CK, purified water at 1 mL kg^−1^ fresh weight (FW)); (ii) AVEO (applied at 1 mL kg^−1^ FW; Baishengyuan Industrial Co., Ltd., Yang Jiang, China); (iii) LB (applied at 1 × 10^6^ CFU/g FW; Zhongke Jiayi Biological Engineering Co., Ltd., Shandong, China); and (iv) VA (applied at 1 mL kg^−1^ FW; Shanghai Macklin Biochemical Co., Ltd., Shanghai, China). All of the samples, weighing 500 g in total, were carefully placed into nylon-polyethylene bags (Deli Group Co., Ltd., Zhejiang, China). These bags were then vacuum-sealed to ensure that all of the air was removed, after which the bags were preserved at a temperature of approximately 25°C. Each treatment included three bags as replicates. Following 60 days of anaerobic fermentation, triticale silage samples were promptly collected from the silage bags of each treatment to analyze fermentation characteristics, aerobic stability, and microbial community composition.

### Aerobic stability analysis of ensiled triticale samples

The ensiled triticale samples, which weighed approximately 3 kg, were transferred into 10 L sterile plastic containers. The silage was not compacted and was left exposed to air at a surrounding temperature of 18–22°C for 7 days. A dual layer of gauze was placed on the surface of the ensiled triticale samples, facilitating the entry of air into the samples. This procedure was used to limit the extent of dust contamination and evaporation that occurred. To obtain temperature readings, a data recorder was positioned in the middle of the silage body. A 7 day aerobic stability test was performed on the triticale silages under a controlled temperature condition of 26.0°C±1.0°C following a 60 day ensiling period (aerobic exposure for 0 days), following the methodology outlined by Okoye et al. (24). According to the definition provided by Schmidt et al. (25), the term "aerobic stability time" refers to the duration of time during which the temperature within the silage remains 2°C above the temperature of the surrounding environment. A real-time temperature recorder that was outfitted with various measurement points was used to obtain this information. To analyze the impacts of air exposure on the pH value, organic acid composition, ammonia-nitrogen (AN) content, and microbial community, samples were collected after 0, 1, 3, 5, and 7 days of fermentation to evaluate the effects of air exposure. To determine the DM, CP, NDF, ADF, starch, hemicellulose, and WSC contents of triticale silage under aerobic fermentation conditions, silage samples were collected and weighed at the beginning and end of the study.

### Chemical composition analysis of ensiled triticale samples

For chemical analysis, fresh triticale samples and triticale silage samples were dried at 65°C for 48 hours until a consistent weight was achieved. This procedure was carried out to determine the amount of DM present in the samples. The anthrone method, which was developed by Owens et al. (26), was utilized to determine the WSC content. The CP content was determined using the Kjeldahl method (12). NDF and ADF determinations were conducted following the methodology of Van Soest et al. (27). The hemicellulose content was calculated by subtracting the NDF and ADF values. After the samples had dried, they were subjected to starch analysis using the methodology outlined by Yuan et al. (28).

### Fermentation composition analysis of ensiled triticale samples

The fermentation properties of freshly harvested triticale and triticale silage were assessed following various periods of aerobic exposure. Briefly, a mixture of 90 mL of sterile water and 10 g of freshly harvested triticale or triticale silage sample that had just been harvested was prepared. Next, the solution was stirred vigorously using a wall breaker for 1 minute, followed by filtration through five layers of gauze. This process was carried out to analyze the levels of organic acids and AN and the pH value. LA, AA, propionic acid (PA), and butyric acid (BA) concentrations were ascertained via high-performance liquid chromatography following the protocols outlined in Jia et al. (29). The pH value was determined with a Raymag pH meter (PHSJ-4F; Yidian Scientific Instrument Co., Ltd., found in Shanghai). The AN concentration in the filtrate was ascertained via the modified phenol-hypochlorite method (30).

### Microbial community composition analysis of ensiled triticale samples

For 3 minutes, each sample filtrate was centrifuged at 10,000 × *g* to extract microbial DNA. The CTAB/SDS technique was used to obtain complete DNA from the genomic regions of the collected samples (28). The amount and purity of the DNA were evaluated via 1% agarose gels. The concentration of DNA was adjusted to 1 µg/μL by adding sterile water. The thermal cycling technique began with an initial denaturation step at 98°C for 1 minute, and the thermal cycling procedure included thirty cycles of denaturation at 98°C for 10 seconds, annealing at 50°C for 30 seconds, and elongation at 72°C for 30 seconds. The sample was subjected to a final temperature of 72°C for 5 minutes. Transcription from the 16S rRNA gene was carried out using the primer pair 806R (5′-GTGGACTACHVGTTWTCTAAT-3′) and 336F (5′-GTACTCCTACGGGAGGCAGCA-3′). The ITS1 forward primer (5′-GTGARTCATCGAATCTTTG-3′) and ITS2 reverse primer (5′-TCCTCCGCTTATTGATATGC-3′) were used to amplify the V9 region of the 18S rRNA gene. We used primers to transcribe the V3–V4 region of the 16S rRNA gene and the V9 region of the 18S rRNA gene. The polymerase chain reaction (PCR) products were combined with an equivalent quantity of 1X loading buffer containing SYBR green. The next step was to conduct electrophoresis on a 2% agarose gel. The PCR products were mixed together in equal quantities. Subsequently, the PCR mixture was purified, and the library was constructed using the TruSeq DNA PCR-Free Sample Preparation Kit. The constructed library was quantified using Qubit and Q-PCR. After confirming the library’s quality, sequencing was performed on the NovaSeq6000 platform. In conclusion, the library produced paired-end reads that were 250 base pairs in length. FLASH software, version VI.2.7, was used to integrate the readings from both ends of the pair. Employing the Uparse algorithm (version 7.0.1001, accessible at http://www.drive5.com/uparse/), all effective tags derived from the samples were grouped into operational taxonomic units (OTUs). For species annotation, the SSU rRNA database from SILVA version 138.1 (available at http://www.arb-silva.de/) was utilized to assign taxonomic classifications to the OTU sequences. Subsequently, the MUSCLE software (version 3.8.31, accessible at http://www.drive5.com/muscle/) facilitated rapid multiple sequence alignment, enabling the determination of phylogenetic relationships among all OTU representative sequences. Ultimately, normalization was performed on the data from each sample. Using Qiime software (Version 1.9.1), the assessment of alpha diversity was subsequently conducted, with particular emphasis on Simpson’s index, Shannon’s index, and Chao 1 richness. The NCBI database already includes the raw sequencing data under accession number PRJNA1213304.

### Data analysis of ensiled triticale samples

Data analysis was accomplished by using the SPSS 26 application (IBM Crop., Armonk, New York, United States). Polynomial orthogonal contrasts (linear and quadratic) were used to determine the response to aerobic exposure at each growth stage of triticale. To perform multiple comparisons among the treatments, Tukey’s test was used. *P* < 0.05 was considered statistically significant. GraphPad Prism software was used to generate graphs of the experimental data.

## RESULTS AND DISCUSSION

### Changes in the chemical composition of ensiled triticale samples during air exposure

Table 1 presents the chemical composition of the triticale silages under aerobic conditions. During the aerobic exposure phase, the concentrations of DM, CP, NDF, ADF, hemicellulose, and starch were considerably lower than those during the ensiling period. Therefore, DM, CP, NDF, ADF, hemicellulose, and starch are consumed and degraded by aerobic microorganisms because of the unrestricted activity of microorganisms in aerobic environments (31). An intriguing phenomenon was observed in the WSC content in the AVEO treatment, in which there was an increase in the WSC content during aerobic exposure, whereas all of the other treatments resulted in a decrease in the WSC content. It is hypothesized that the greater relative abundance of *Bacillus* in AVEO-treated silage (Fig. 2B) in the presence of oxygen increased amylase secretion, promoting the breakdown of starch and consequently increasing the WSC content (32). In contrast, an increase in hemicellulose conversion was shown to be responsible for a part of the increase in the WSC content that was detected in the AVEO treatment (7). Further research is needed to identify the specific active ingredient responsible for the accelerated degradation of starch. Throughout the 7 days of aerobic exposure, the LB treatment resulted in greater concentrations of DM and starch than did the CK treatment, whereas the NDF level was relatively low. In the AVEO treatment, the levels of DM, CP, starch, and WSC increased, whereas the NDF, ADF, and hemicellulose contents considerably decreased in comparison with those in the CK treatment group. In addition, the DM concentrations were greater in the VA treatment, whereas the NDF and hemicellulose concentrations were significantly lower than those in the CK treatment. Because of its capacity to suppress a wide variety of bacteria, AVEO is able to prevent aerobic microorganisms from breaking down substances such as DM, CP, and WSC (23). Likewise, the presence of organic acids during AVEO treatment leads to the breakdown of lignocellulosic components, including NDF, ADF, and hemicellulose, throughout the process (9). The decrease in starch content observed in the AVEO treatment group corresponded to an increase in the WSC content, which may be related to the greater relative abundance of *Bacillus* in the AVEO treatment group (Fig. 2B) and the secretion of more amylase in the AVEO treatment group than in the CK group (32). The acid-producing capacity of LB also led to increased levels of DM and starch in the LB treatment group, which aligns with the conclusions of Zhang et al. (12) in their investigation of native grass silage. VA, an acidic compound, can decrease the utilization of DM through the inhibition of the proliferation of other bacterial species. Furthermore, its acidic characteristics facilitate the decomposition of NDF and hemicellulose (15).

**TABLE 1:** Chemical composition of triticale silage fermented with different additives during aerobic exposure^*a*^^,*b*,*c*^

^Items^	Treatment (T)	Aerobic exposure period (D)		SEM	*P* value		
		Day 0	Day 7		T	D	T × D
DM	CK	58.56Ab	49.46Bb				
%FM	AVEO	67.98Aa	63.40Ba	0.278	<0.001	<0.001	<0.001
	LB	60.80Aa	56.40Ba				
	VA	61.16Ab	55.88Ba				
CP	CK	6.32Ac	6.25Bb				
%DM	AVEO	7.80Aab	7.46Ba	0.060	<0.001	<0.001	<0.001
	LB	6.94Ab	6.39Bb				
	VA	8.20Aa	6.71Bb				
NDF	CK	65.94Aa	61.93Ba				
%DM	AVEO	58.30Ab	52.09Bc	0.394	<0.001	<0.001	0.548
	LB	61.47Ab	57.36Bb				
	VA	62.00Aab	56.33Bb				
ADF	CK	43.08Aa	40.35Ba				
%DM	AVEO	39.88Ab	35.81Bb	0.459	0.002	<0.001	0.366
	LB	40.13Aab	38.48Ba				
	VA	41.99Aab	39.98Ba				
Hemicellulose	CK	22.86A	21.58Ba				
%DM	AVEO	18.42A	16.28Bb	0.680	0.022	0.050	0.958
	LB	21.34A	18.52Bab				
	VA	20.01A	17.85Bb				
Starch	CK	21.76Ab	19.48Bb				
%DM	AVEO	24.07Aa	18.63Bc	0.240	<0.001	<0.001	0.001
	LB	23.07Aab	21.50Ba				
	VA	22.47Ab	20.33Bb				
WSC	CK	2.21Ab	1.72Bb				
%DM	AVEO	3.13Ba	5.11Aa	0.042	<0.001	0.048	<0.001
	LB	2.88Aa	2.33Bb				
	VA	2.91Aa	2.36Bb				

^*a*^ CK, control group (no exogenous inoculant); AVEO, *Amomum villosum* essential oil; LB, inoculation of *L. buchneri*; VA, vanillic acid; A–D different superscript letters in mean values reported in the column pertaining to aerobic exposure days indicate differences among samples of the same treatment (*P* < 0.05).

^*b*^ Different superscript letters in mean values reported in a single row pertaining to different treatment groups indicate differences among sample groups based on aerobic exposure day (*P* < 0.05); DM, dry matter; CP, crude protein; NDF, neutral detergent fiber; ADF, acid detergent fiber; WSC, water-soluble carbohydrate; Day 0, 60 days of ensiling (aerobic exposure for 0 days); Day 7 corresponds to 7 days of aerobic exposure. T, treatments; D, days of aerobic exposure; T × D, interaction between days of aerobic exposure and treatments; SEM, standard error of the mean.

^*c*^ Empty cells indicate that the *P*-values and/or standard errors could not be calculated due to insufficient data or non-applicable values for the given indicator.

### Fermentation characteristics and aerobic stability of ensiled triticale samples during air exposure

The characteristics of fermentation that were observed in triticale silage samples that were exposed to aerobic conditions are detailed in Table 2. Over the course of this investigation, the pH value and LA, AA, and AN concentrations were found to be substantially influenced by the treatment and the interactions between the treatment and aerobic exposure days (*P* < 0.05). The pH value quadratically (*P* < 0.01) increased with increasing aerobic exposure days, and the LA and AN concentrations linearly (*P* < 0.01) decreased with increasing aerobic exposure days. All of the samples presented increases in the pH and AN content, whereas the LA content steadily increased as the duration of aerobic exposure increased. Previous research has shown that aerobic exposure induces an increase in pH and a reduction in the LA concentration within silage (33). This phenomenon is attributed to the activation of aerobic bacteria and yeast upon contact with air, whose metabolic activities degrade and consume LA, ultimately leading to an increase in pH (33). On the basis of the findings of Mugabe et al. (34), the gradual exposure of anaerobically fermented silage to oxygen facilitates the conversion of various proteins and soluble carbohydrates into AN by aerobic microbiota. However, an increase in pH diminishes the suppressive influence of the acidic milieu on the proliferative activity of aerobic microorganisms, thereby contributing to an increase in the AN concentration. The AA content decreased in the AVEO, LB, and CK treatment groups after 5 days of aerobic exposure as aerobic exposure progressed. This demonstrated the initiation and progression of aerobic spoilage in triticale silages, as well as the varying levels of aerobic stability observed in the different treatment groups. Following 60 days of ensiling (aerobic exposure for 0 days), the groups treated with AVEO, LB, or VA presented higher LA contents than did those in the CK treatment. The outcomes of the present investigation align with those of prior research on the ensiling of paper mulberry (23), whole-crop corn (33), and stylo (15), which revealed that the LA concentration in the additive-treated groups (AVEO, LB, and VA) was notably elevated compared with that in the CK treatment group. The groups treated with LB presented greater AA contents than did those in the CK treatment group. This is attributed to the fact that the LB-treated group underwent heterofermentative fermentation metabolism during ensiling, resulting in the coproduction of LA and AA, which may consequently increase the concentration of AA (24). After 60 days of ensiling (aerobic exposure for 0 days), the groups treated with AVEO and LB presented lower AN contents than did those in the CK treatment group. The findings of the present study corroborate those of the research on the ensiling of paper mulberry (23) and corn stalk (24), demonstrating that the AN concentration in the additive-treated groups (AVEO and LB) was significantly lower than that in the CK group. Following 7 days of aerobic exposure, the groups treated with AVEO, LB, or VA presented lower pH values and AN levels than did those in the CK treatment group. Nevertheless, they presented higher LA and AA levels than did the CK treatment group. While the AVEO treatment group had the greatest LA concentration and the lowest pH value, it also had the lowest AN content. Moreover, the LB and AVEO treatment groups had the highest AA contents. The observed phenomenon can be attributed to the increase in the relative abundance of *Lactiplantibacillus* within the triticale silage following the addition of AVEO, which subsequently led to elevated metabolic activity of these populations, contributing to a significant increase in the LA concentration and a delayed increase in the pH value within the AVEO-treated group (23). Owing to the greater relative abundance of LAB in the AVEO treatment and the reduced consumption of LA compared with the LB treatment group, the AVEO treatment resulted in the highest LA content and the lowest pH value. Furthermore, AVEO is composed of phenolic compounds and terpenoids, including lobsteryl acetate, camphorene, and D-limonene, which exhibit inhibitory effects on various fungi, including yeasts and molds, hence reducing the breakdown of proteins into AN (22). Furthermore, AVEO is predominantly composed of lobsteryl acetate, which undergoes hydrolysis to form lobsteryl acid and AA in the presence of water-soluble acids, resulting in a greater concentration of AA in triticale silage samples (35). Owing to the antifungal substances inherently present in AVEO, along with their high contents of LA and AA, it strongly inhibits activity of various fungi, including yeasts and molds, thus leading to the lowest AN content (22).

**TABLE 2:** The fermentation characteristics of triticale silage fermented with different additives after 60 days of ensiling and during aerobic exposure^*a*^^,*b*,*c*^

Items	Treatment (T)	Aerobic exposure period (D)					SEM	*P* value			
		Day 0	Day 1	Day 3	Day 5	Day 7		T	L	Q	T × D
pH	CK	3.93B	3.94B	4.03Ba	4.10B	6.56Aa			<0.001	<0.001	
	AVEO	3.89D	3.92 CD	3.97BCb	3.97B	4.19Ab	0.018	<0.001	<0.001	0.002	<0.001
	LB	3.91C	3.92C	3.99Bab	4.00B	4.38Ab			<0.001	<0.001	
	VA	3.91B	3.93B	4.00Bab	4.01B	4.84Ab			0.001	0.019	
LA	CK	2.36Ab	2.59Ab	2.21ABb	1.86Bb	0.33Cb			<0.001	<0.001	
%DM	AVEO	6.40Aa	5.10ABa	4.04BCa	2.99Ca	1.91 Da	0.060	<0.001	<0.001	0.950	<0.001
	LB	4.81Aa	4.43Aa	3.22Bab	2.65Ba	1.09Ca			<0.001	0.178	
	VA	5.53Aa	3.92ABab	3.48Ba	2.52BCa	0.92Ca			<0.001	0.970	
AA	CK	1.34Ab	1.46A	1.68Ab	0.82ABb	0.05Bb			0.001	0.016	
%DM	AVEO	1.98Ab	2.07A	2.08Ab	2.24Aab	0.92Ba	0.071	<0.001	0.001	0.001	0.033
	LB	3.39Aa	3.24A	3.69Aa	2.95Aa	0.92Ba			0.001	0.003	
	VA	1.05b	2.43	1.31b	1.92ab	0.81 a			0.362	0.159	
PA	CK	0.69	1.29	1.50	1.27	1.69					
%DM	AVEO	/^*d*^	/	/	/	/					
	LB	/	/	/	/	/					
	VA	/	/	/	/	/					
AN	CK	0.49 Da	0.62 CD	0.82Ca	1.08Ba	1.84Aa			<0.001	<0.001	
%DM	AVEO	0.37 Cc	0.55B	0.61ABb	0.68ABb	0.72Ac	0.011	<0.001	<0.001	0.029	<0.001
	LB	0.40Dbc	0.58 CD	0.76BCab	0.95ABab	1.09Ab			<0.001	0.315	
	VA	0.44Cab	0.54 CD	0.74BCab	0.97ABa	1.19Ab			<0.001	0.619	

^*a*^ CK, control group (no exogenous inoculant); AVEO, *Amomum villosum* essential oil; LB, inoculation of *L. buchneri*; VA, vanillic acid; A–D different superscript letters in mean values reported in the column pertaining to aerobic exposure days indicate differences among samples of the same treatment group (*P* < 0.05).

^*b*^ Different superscript letters in mean values reported in a single row pertaining to different treatment groups indicate differences among sample groups on the basis of aerobic exposure day (*P* < 0.05); LA, lactic acid; AA, acetic acid; PA, propionic acid; AN, ammonia nitrogen; ND, not detected; Day 0, 60 days of ensiling (aerobic exposure for 0 days); Days 1, 3, 5 and 7 correspond to 1, 3, 5 and 7 days of aerobic exposure, respectively. T, treatments; D, days of aerobic exposure; L and Q represent linear and quadratic effects of aerobic exposure time, respectively; T × D, interaction between days of aerobic exposure and treatments; SEM, standard error of the mean.

^*c*^ Empty cells indicate that the *P*-values and/or standard errors could not be calculated due to insufficient data or non-applicable values for the given indicator.

^*d*^ "/", indicates index was not detected.

During the silage fermentation process in the LB-treated group, heterofermentative metabolism was induced, leading to the concurrent production of LA and AA and, consequently, an increase in their respective concentrations (24). The resulting acidic milieu, attributed to the elevated levels of LA and AA, effectively suppressed the proliferation of detrimental microorganisms, retarded the increase in pH, and impeded protein degradation, ultimately resulting in decreased AN production (36). The addition of the acidic compound VA, which exhibits acidic, antibacterial, and enzymatic inhibitory characteristics, reduced the pH value of VA treatment, accelerated the establishment of LAB dominance, stimulated an increase in the LA content, and potentially mediated its effects through the inhibition of protease activity and microbial deaminase activity, ultimately leading to a decrease in the production of AN (15). The outcomes of the present investigation align with those of prior research on the ensiling of stylo (15), which revealed that the AN concentration in the VA-treated groups was notably lower than that in the CK treatment group.

**Fig 1:**
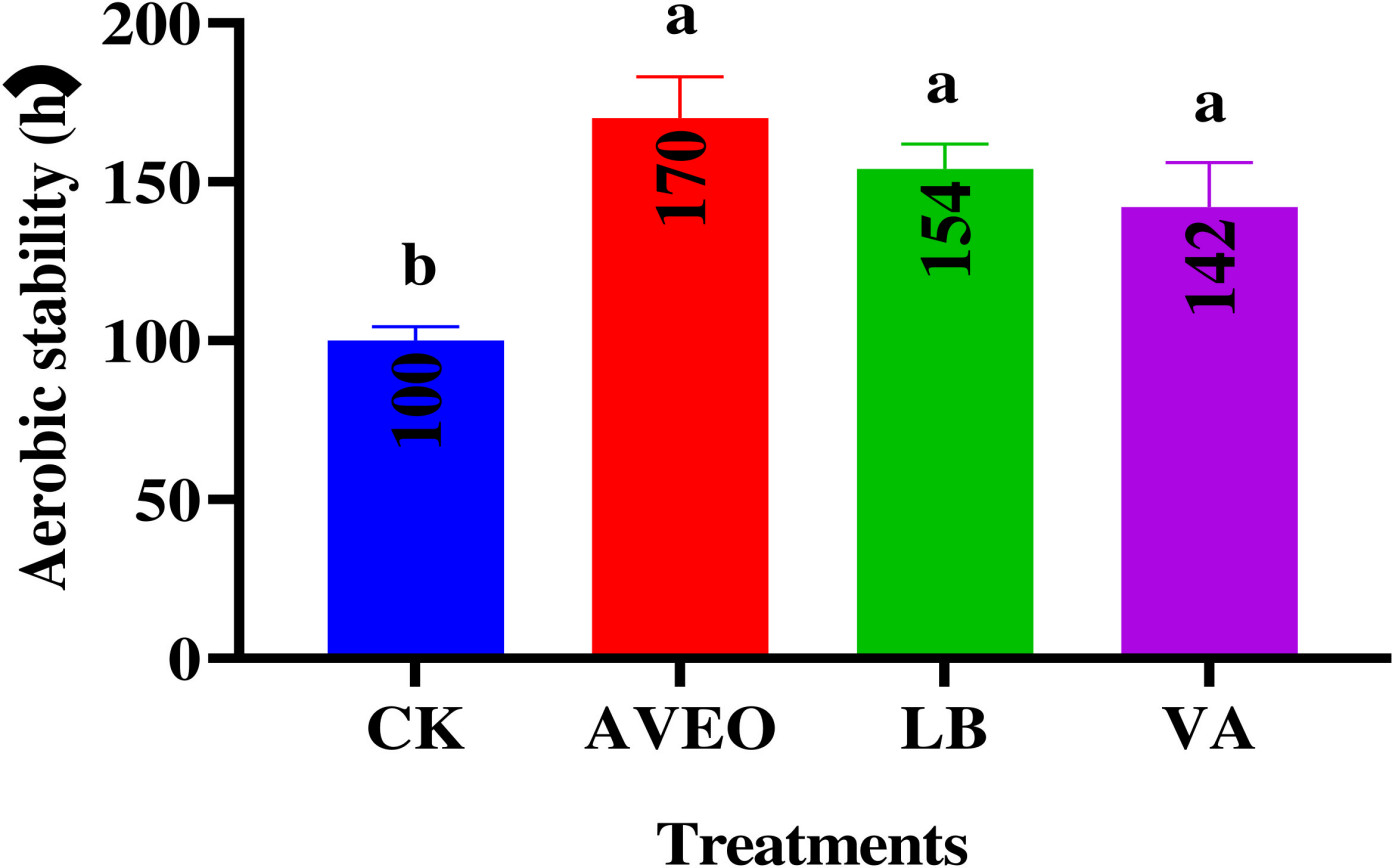
Aerobic stability of triticale silage during aerobic exposure. CK, control group (no exogenous inoculant); AVEO, *Amomum villosum* essential oil; LB, inoculation with *L. buchneri*; VA, vanillic acid.

The aerobic stability of the ensiled triticale samples is demonstrated in Fig. 1. Compared with the CK treatment, the AVEO treatment resulted in greater aerobic stability, followed by LB and VA treatments. When LB is employed as an additive, it notably has a superior inhibitory effect on aerobic spoilage and prolongs the duration of silage preservation under aerobic conditions, a result that has been demonstrated by Okoye et al. (24). Previous studies have reported comparable findings, indicating that the incorporation of LB into *Leymus chinensis* silage significantly increases its AA concentration and enhances its aerobic stability (36).

Despite having a lower level of AA than that in the LB treatment group, the AVEO treatment group exhibited similar aerobic stability to the LB treatment and even surpassed it in duration. The observed difference can be ascribed to the presence of potent antibacterial compounds in AVEO, specifically lobsteryl acetate, camphorene, and D-limonene, which exert powerful inhibitory effects, thereby enabling the AVEO-treated group to effectively suppress the proliferation of aerobic microorganisms throughout the entire aerobic exposure period (22, 37). As a result, AVEO treatment is able to produce optimal aerobic stability. The aerobic stability period observed in the AVEO-treated triticale silage in our study (170 h) surpassed the maximum aerobic stability duration (99.76 h) reported by Li et al. (4) in their investigation of the aerobic stability characteristics of triticale silage, indicating that the additive we used is more conducive to enhancing the aerobic stability of triticale silage.

### Microbial diversity of ensiled triticale samples during air exposure

Table 3 displays the microbial diversity of the triticale silage when exposed to air. For bacteria, the Chao 1 and Shannon indices (*P* < 0.05) of the triticale silage samples progressively increased after 5 days of aerobic exposure. For fungi, the Chao 1, Simpson, and Shannon indices (*P* < 0.05) decreased progressively in all the triticale silage samples after 5 days of aerobic exposure. These findings indicate that the presence of oxygen within the environment led to a decrease in LAB dominance, facilitated aerobic bacterial development, and enhanced bacterial community diversity. In contrast, fungal diversity decreased under aerobic conditions, potentially due to the dominance of certain species within the fungal community. The outcomes of the present investigation align with the findings published by Li et al. (38) regarding forage oat silage. The silage treated with additives during aerobic exposure had the lowest bacterial diversity index, mostly attributed to its acidic pH, which hindered microbial development and hence resulted in reduced bacterial variety (38). Nevertheless, the fungal diversity index was similarly reduced in the additive treatment group, possibly due to the inhibitory effect of AA on fungal colonization. Furthermore, research has shown that the proliferation of dominant bacteria in silage leads to a reduction in the overall variety of bacterial populations (36).

**TABLE 3:** The microbial diversity of bacteria in triticale silage fermented with different additives after 60 days of ensiling and during aerobic exposure^*a*^^,^^*b*^^,^^*c*^

	Items	Treatment (T)	Aerobic exposure period (D)					SEM	*P* value			
			Day 0	Day 1	Day 3	Day 5	Day 7		T	L	Q	T × D
		CK	504.22D	546.59 CD	617.85C	730.86Ba	893.26Aa			<0.001	0.042	
	Chao1	AVEO	428.68B	424.36B	471.70B	539.47ABb	635.59Ab	6.713	<0.001	<0.001	0.147	0.220
		LB	462.71B	451.91B	490.15AB	555.47ABb	673.40Ab			0.002	0.174	
		VA	479.78B	483.36B	511.60B	581.41ABb	688.69Ab			<0.001	0.070	
		CK	0.94	0.96	0.97 a	0.97	0.97			0.097	0.309	
Bacteria	Simpson	AVEO	0.82B	0.91AB	0.91ABb	0.94A	0.94A	0.004	0.001	0.004	0.099	0.611
		LB	0.90	0.93	0.94ab	0.94	0.96			0.028	0.488	
		VA	0.90	0.92	0.93ab	0.95	0.95			0.218	0.743	
		CK	5.76Ba	6.07AB	6.27ABa	6.47ABa	6.92A			0.003	0.881	
	Shannon	AVEO	4.86Cb	5.00BC	5.38ABCb	5.63ABb	6.12A	0.040	<0.001	<0.001	0.694	0.884
		LB	5.27Bab	5.42B	5.45Bb	5.99ABab	6.46A			<0.001	0.160	
		VA	5.45Bab	5.67B	5.68Bb	5.74Bb	6.70A			<0.001	0.032	
		CK	325.57Aa	307.37Aa	296.30Aa	218.03B	139.93Ca			<0.001	0.017	
	Chao1	AVEO	239.20Ab	221.12Ab	208.49Ab	200.41A	56.11Bb	3.470	<0.001	<0.001	0.001	0.192
		LB	252.14Aab	244.27Aab	232.56Ab	212.87A	116.31Bab			<0.001	0.020	
		VA	268.47Aab	252.49Aab	225.77Ab	205.95AB	137.85Ba			<0.001	0.313	
		CK	0.96Aa	0.93Aa	0.88A	0.76AB	0.57B			<0.001	0.097	
Fungi	Simpson	AVEO	0.84Ab	0.80Ab	0.80A	0.70A	0.38B	0.011	0.007	<0.001	0.016	0.998
		LB	0.90Aab	0.87Aab	0.82A	0.73A	0.48B			<0.001	0.065	
		VA	0.91Aab	0.88Aab	0.82A	0.74A	0.50B			<0.001	0.053	
		CK	6.52A	5.99A	5.27AB	4.54B	2.75C			<0.001	0.141	
	Shannon	AVEO	6.09A	5.67AB	4.76BC	4.01C	1.76D	0.063	0.028	<0.001	0.014	0.996
		LB	6.21A	5.66AB	4.82AB	4.29B	2.37C			<0.001	0.208	
		VA	6.36A	5.71AB	4.80BC	4.34C	2.45D			<0.001	0.127	

^*a*^ CK, control group (no exogenous inoculant); AVEO, *Amomum villosum* essential oil; LB, inoculation of *L. buchneri*; VA, vanillic acid; A–D different superscript letters in mean values reported in the column pertaining to aerobic exposure days indicate differences among samples of the same treatment group (*P* < 0.05).

^*b*^ Different superscript letters in mean values reported in a single row pertaining to different treatment groups indicate differences among sample groups on the basis of aerobic exposure day (*P* < 0.05); Day 0, 60 days of ensiling (aerobic exposure for 0 days); Days 1, 3, 5, and 7 correspond to 1, 3, 5, and 7 days of aerobic exposure, respectively. T, treatments; D, days of aerobic exposure; L and Q represent linear and quadratic effects of aerobic exposure time, respectively; T × D, interaction between days of aerobic exposure and treatments; SEM, standard error of the mean.

^*c*^ Empty cells indicate that the *P*-values and/or standard errors could not be calculated due to insufficient data or non-applicable values for the given indicator.

The bacterial diversity in the AVEO-treated group was the lowest due to the promotion of LAB, the dominant bacterial flora, by AVEO, thereby resulting in a decrease in overall bacterial diversity (23). In contrast, the observed decrease in fungal diversity in the AVEO treatment group can be attributed to the presence of additional active antifungal components, which hinder the growth and reproduction of fungi, resulting in reduced fungal diversity (21).

### Microbial community composition of ensiled triticale samples during air exposure

**Fig 2:**
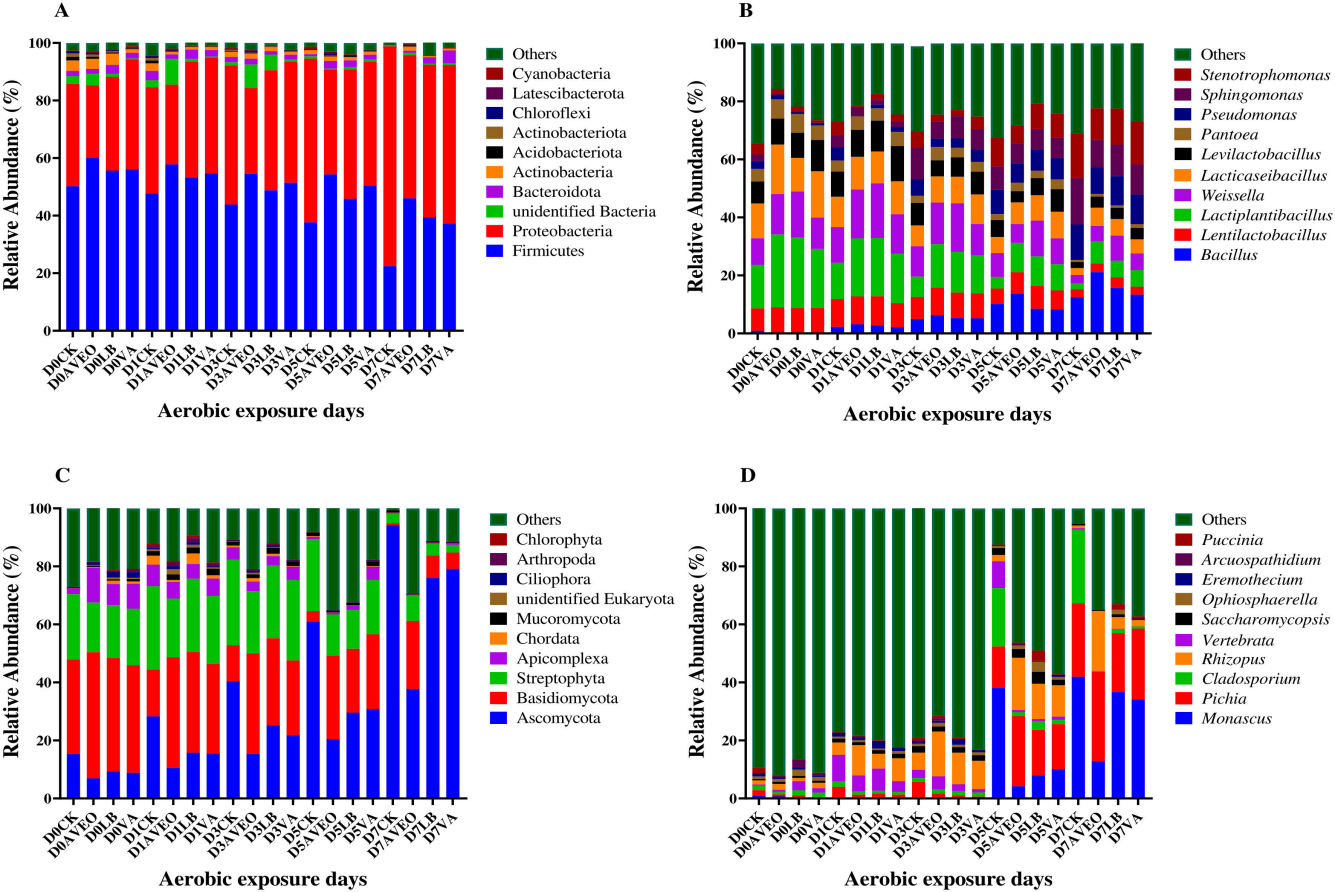
Microbial communities of triticale silage after 60 days of ensiling (aerobic exposure for 0 days) and during aerobic exposure. The bacterial communities are shown at the phylum level (**A**) and genus level (**B**). The fungal communities are shown at the phylum level (**C**) and genus level (**D**). CK, control group (no exogenous inoculant); AVEO, *Amomum villosum* essential oil; LB, inoculation of *L. buchneri*; VA, vanillic acid.

The taxonomic classification of the bacterial population at the phylum level varied, as demonstrated in Fig. 2A. Among all of the triticale silage samples collected during the 60 days of ensiling (aerobic exposure for 0 days) and throughout the 7-day aerobic exposure period, the dominant phyla that were identified were Firmicutes and Proteobacteria. Our findings align with the bacterial community composition of triticale silage after 60 days of ensiling (aerobic exposure for 0 days), as reported by Li et al. (4), and are also consistent with the bacterial community structure observed in *L. chinensis* silage during the aerobic exposure period, as described by Wu et al. (36). The relative abundance of Firmicutes decreased, and the relative abundance of Proteobacteria progressively increased with increasing duration of aerobic exposure, corroborating the findings of Wu et al. (36) in their investigation of *L. chinensis* silage. However, Firmicutes consistently remained at a high level, surpassing that of the CK treatment in all additive treatments, with the maximum relative abundance observed in the AVEO treatment group. At the same time, Proteobacteria remained at a level below that of the CK treatment group across all additive treatment groups, particularly the AVEO treatment group, which presented the lowest relative abundance. As verified by Liu et al. (39), the relative abundance of Firmicutes remained high, possibly due to the presence of facultative anaerobic bacteria and some aerobic bacteria within the Firmicutes phylum, accounting for the higher abundance observed in the additive-treated group. In the additive treatment, the observed gradual increase in the relative abundance of Proteobacteria could be linked to an increase in pH, where the mitigation of acidic conditions activates certain harmful bacteria, thereby enabling their growth and reproduction (1, 7). In the AVEO treatment group, a relatively high abundance of LAB belonging to Firmicutes was observed concurrently with the effective inhibition of harmful bacterial growth and reproduction by the presence of LA, AA, and other antibacterially active substances, thereby preserving the relative abundance of Firmicutes and hindering the increase in the relative abundance of Proteobacteria (22, 35). As demonstrated in Fig. 2B, the predominant genus identified in all 60-day ensiling (aerobic exposure for 0 days) triticale samples was *Lactiplantibacillus*, which aligns with the results published by Liu et al. (39). As the duration of aerobic exposure increased, the relative abundance of LAB in each silage treatment decreased, whereas the relative abundances of *Bacillus*, *Sphingomonas*, and *Stenotrophomonas* increased. Owing to the anaerobic nature of LAB, their relative abundance decreases under aerobic conditions, whereas *Bacillus* can thrive in an aerobic environment, increasing their relative abundance (40). The literature indicates that *Sphingomonas* and *Stenotrophomonas* are present during the aerobic exposure phase, and consequently, their relative abundance increases as the duration of aerobic exposure increases (41, 42). The samples in the additive treatment groups presented greater relative abundances of *Bacillus* and *Lactiplantibacillus* than did those in the CK treatment group after 7 days of aerobic exposure. Conversely, compared with the CK treatment, the additive treatment resulted in reduced abundances of *Sphingomonas* and *Stenotrophomonas*. In contrast, the AVEO treatment resulted in lower relative abundances of *Stenotrophomonas* and *Sphingomonas*. Compared with the CK group, the LB-treated group presented greater aerobic stability, which was in line with the findings of prior studies (24, 43). Additionally, the presence of AA in the LB treatment group inhibited the growth of *Stenotrophomonas* and *Sphingomonas* (43). Notably, in contrast with the other treatment groups, the LB treatment group presented no significant increase in the relative abundance of *Lentilactobacillus*. This is because homofermentative LAB exhibit superior adaptability to acidic environments, conferring a competitive edge during the later stages of fermentation (44). In contrast, the heterofermentative LAB (*Lentilactobacillus*) fails to accommodate acidic conditions and is subsequently outcompeted, leading to a decline in its abundance. In the VA treatment group, the acidity of VA mitigated the increase in pH, consequently impeding the substitution of *Lactiplantibacillus* and suppressing the proliferation of acid-sensitive bacteria, including *Sphingomonas* and *Stenotrophomonas* (36, 44). The capacity of AVEO to stimulate the development of specific facultative anaerobic LAB may explain why there was a markedly greater abundance of *Lactiplantibacillus* in the AVEO treatment than in the other treatment groups (45), maintaining an acidic environment and slowing the substitution of LAB. Furthermore, the presence of AA and other antibacterial components in the AVEO treatment restricted the growth of harmful bacteria such as *Sphingomonas* and *Stenotrophomonas* (23). In addition, research conducted by Bai et al. (46) revealed that *Bacillus* species may improve the aerobic stability of silage samples. Our results support this finding as the treatment group with the highest aerobic stability also had the highest relative abundance of *Bacillus*. However, the specific mechanism by which AVEO enriches *Bacillus* is still unknown and requires further investigation.

In summary, aerobic exposure reduced the proportion of *Lactiplantibacillus* in triticale silage, and AVEO helped maintain a high relative abundance of *Lactiplantibacillus* and *Bacillus* after 7 days of aerobic exposure, suggesting that AVEO acts as an enrichment agent for *Lactiplantibacillus* and *Bacillus* in triticale silage.

The fungal community at the phylum level varied, as shown in Fig. 2C. The most prevalent phyla in the triticale silage samples subjected to the ensiling and aerobic exposure phases were Ascomycota and Basidiomycota, respectively. This finding aligns with the alterations observed in the fungal phylum composition of corn silage, as reported by Yin et al. (33). Since the duration of aerobic exposure increased, there was a consistent increase in the relative abundance of Ascomycota. Additionally, the presence of additives consistently reduced the relative abundance of Ascomycota in all the treatment groups, especially in the AVEO treatment group, which presented the lowest relative abundance. This finding corresponds with the alterations observed in the fungal phylum composition of sugarcane top silage, as documented by Zhang et al. (47). The relative abundance of Basidiomycota decreased progressively with longer periods of aerobic exposure. These findings are consistent with the changes in the fungal phylum composition of *L. chinensis* silage, as reported by Liu et al. (44). Moreover, Basidiomycota consistently exceeded that in the CK treatment in all of the treatments with additives, reaching its peak in the AVEO treatment. Ascomycota was less abundant in the AVEO-treated samples than in the other treatment samples, possibly because of the better aerobic stability in the AVEO-treated samples.

The fungal community at the genus level varied, as illustrated in Fig. 2D. The dominant fungal genera were *Vertebrata* and *Rhizopus* before 3 days of aerobic exposure of triticale silage, and the dominant fungal genera were *Monascus*, *Pichia*, and *Rhizopus* in the additive treatment groups and *Monascus*, *Pichia*, and *Cladosporium* in the CK treatment group after 3 days of aerobic exposure. The relative abundances of *Monascus*, *Pichia*, and *Cladosporium* increased, whereas the relative abundance of *Rhizopus* decreased with the increasing duration of aerobic exposure in each of the triticale silages. Liu et al. (44) and Jiang et al. (48) examined the occurrence of *Monascus* in the aerobic stability of *L. chinensis* and alfalfa silage, respectively. Researchers have discovered that silage with weaker aerobic stability has a greater relative abundance of *Monascus* than other kinds of silage (44). Furthermore, correlation analyses revealed a significant association between the presence of *Monascus* and the occurrence of aerobic deterioration. *Pichia* has been reported in studies investigating the aerobic stability of both forage oat (38) and alfalfa (46) silage, and it is one of the most frequently detected fungi in aerobically spoiled silage. This is because fungal succession in silage is usually initiated by yeasts with elevated pH values after exposure to air, which accelerates the proliferation of certain acid-intolerant and detrimental microorganisms (49). In contrast, *Pichia* can metabolize organic acids and increase the pH value because of its ability to take up LA (50). Therefore, there is a strongly significant connection to the aerobic corruption of silage.

*Cladosporium* has been detected in investigations on the aerobic stability of sugarcane silage as well as fermented soybean meal (47, 51), where it was linked to the formation of mycotoxins. Thus, the relative abundances of *Monascus, Pichia,* and *Cladosporium* increased progressively with aerobic exposure. Nevertheless, *Rhizopus* species exhibit remarkable glycolytic enzyme production capabilities and can utilize a wide range of carbohydrates to generate LA, phenolic compounds, and other essential nutrients in aerobic environments (52). After 7 days of aerobic exposure, the relative abundance of *Rhizopus* was greater in the additive treatment groups than in the CK treatment group, whereas the relative abundances of *Monascus* and *Cladosporium* were less prevalent in the additive treatment groups than in the CK treatment groups. Additionally, the relative abundance of *Rhizopus* was relatively high, and those of *Monascus* and *Cladosporium* were relatively low in the AVEO treatment group. Our research results align with the observations of Liu et al. (44), who reported a decrease in the relative abundance of *Monascus* within *L. chinensis* silage upon the incorporation of LB. The LB and VA treatments inhibited fungal growth and reduced the relative abundances of *Monascus* and *Cladosporium*, which may be attributed to the presence of AA. In addition to AA, which inhibited fungal growth, the AVEO treatment group also presented a greater relative abundance of *Rhizopus*, resulting in the continuous generation of LA under aerobic conditions (52). This led to the maintenance of an acidic environment, inhibited fungal proliferation, and delayed aerobic deterioration.

### Correlations of microbial communities with ensiling characteristics in ensiled triticale samples during air exposure

**Fig 3:**
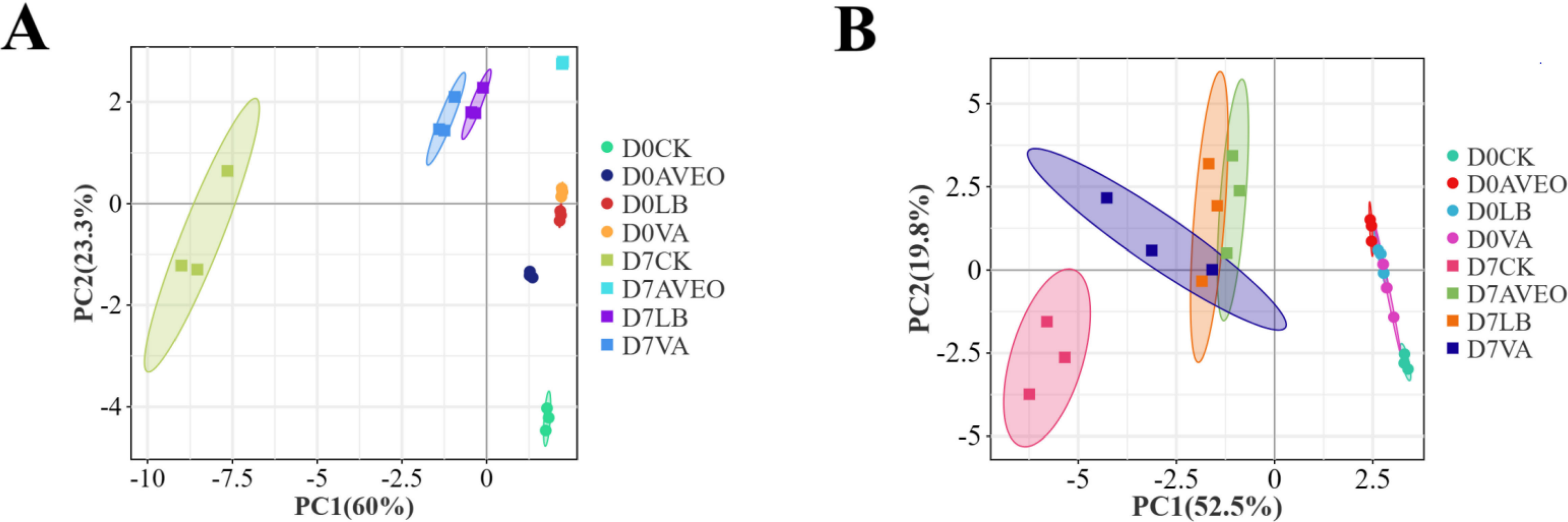
Principal coordinate analysis (PCoA) plots of the operational taxonomic units (OTUs) of the bacterial (**A**) and fungal (**B**) communities in triticale silage after 60 days of ensiling (aerobic exposure for 0 days) and 7 days of aerobic exposure. CK, control group (no exogenous inoculant); AVEO, *Amomum villosum* essential oil; LB, inoculation of *L. buchneri*; VA, vanillic acid.

Fig. 3A and B shows the results of the principal coordinate analysis (PCoA) conducted on the levels of microbial OTUs in triticale silage after 0 and 7 days of aerobic exposure. The bacterial and fungal communities of the triticale silage samples after 0 days of aerobic exposure were significantly separated from those of the triticale silage samples after 7 days of exposure to air. This phenomenon could be used to trace the changes that occur in the bacterial and fungal communities as a result of the shift from anaerobic to aerobic circumstances. These findings align with the findings reported in the study performed by Yin et al. (33) regarding the aerobic stability of maize silage. There was always a clear distinction between the bacterial and fungal communities of the additive and CK treatment groups throughout the aerobic exposure phase. This observation suggested that the additives induced alterations in the bacterial and fungal communities, which had beneficial implications for the quality and aerobic stability of the silage in the treated groups.

**Fig 4:**
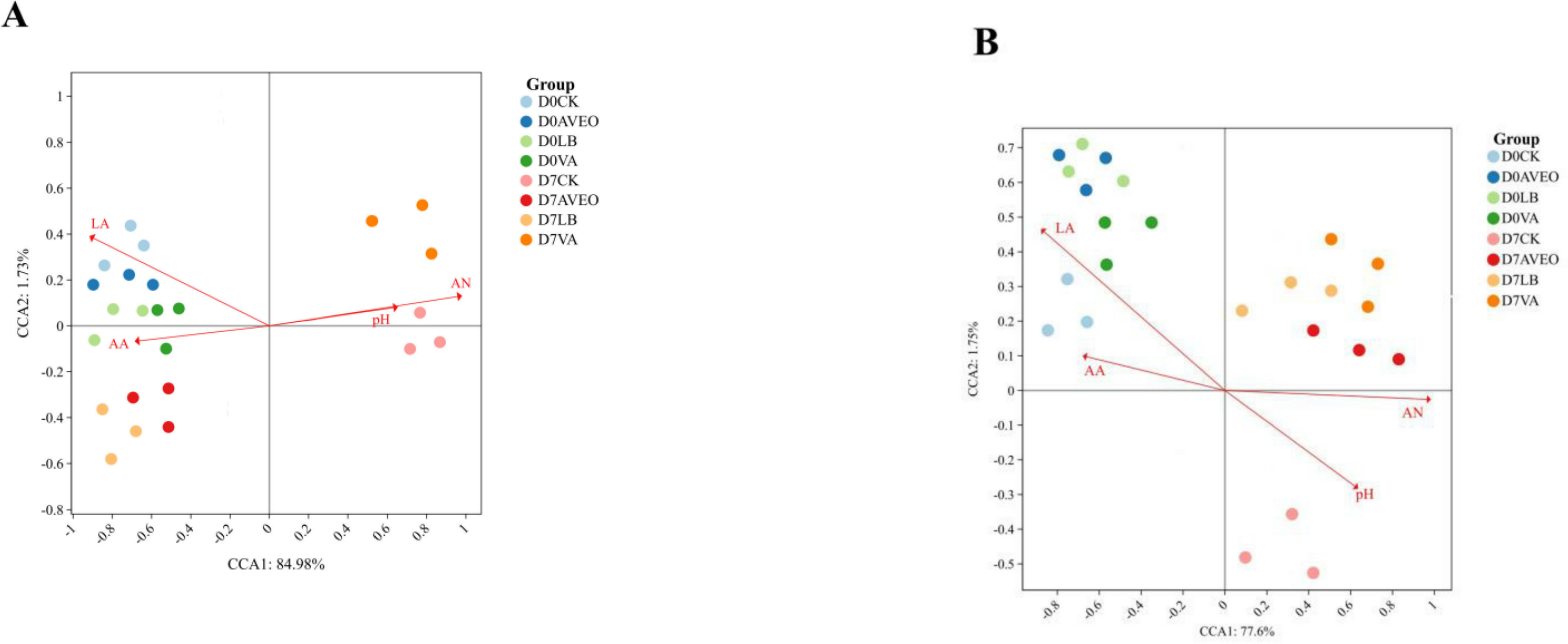
Canonical correlation analysis (CCA) of the operational taxonomic units (OTUs) of the bacterial (**A**) and fungal (**B**) communities in triticale silage after 60 days of ensiling (aerobic exposure for 0 days) and 7 days of aerobic exposure. CK, control group (no exogenous inoculant); AVEO, *Amomum villosum* essential oil; LB, inoculation of *L. buchneri*; VA, vanillic acid.

Fig. 4A and B shows the results of canonical correlation analysis (CCA) applied to evaluate the connections between the four treatment groups (CK, AVEO, LB, and VA treatments) and four environmental parameters (pH and LA, AA, and AN contents). In the bacterial community, after 0 days of aerobic exposure, a positive correlation was observed between the concentrations of LA and AA in all of the treatment groups. On the other hand, a negative correlation was observed between the AN content and pH value in all of the treatment groups. This occurred because, in anaerobic environments, the main bacteria in all of the treatment groups were homofermentative and heterofermentative LAB. The constant production of LA and AA by these bacteria led to a reduction in the pH and a slowdown in the production of AN (38), which is a normal tendency in the fermentation process of silage. After 7 days of aerobic treatment, the LA and AA contents were positively correlated with the AVEO and LB treatments but negatively correlated with the CK treatment. The AN concentration and pH were positively correlated with the CK treatment, whereas they were negatively correlated with the AVEO and LB treatments. Aerobic exposure in the CK treatment group led to an increased pH value, and these samples contained LA and AA, which have been shown to be consumed and utilized by other aerobic bacteria (33). Moreover, an aerobic environment favors the propagation of *Clostridium* spp., which increases the consumption of CP. Therefore, the AN content is elevated (34). Moreover, AVEO could impede the consumption of LA and AA by aerobic bacteria and the breakdown of CP by *Clostridium* because of its strong ability to inhibit bacterial activity (23). LB treatment reduced the consumption of LA and AA and the production of AN due to the increased AA content, which inhibited aerobic bacterial activity to some extent (12). A positive correlation was identified in the fungal community between the concentrations of LA and AA present in each treatment following 0 days of aerobic exposure. The AN content and pH value were negatively correlated with each treatment. In an anaerobic environment, most fungi are suppressed, and bacteria dominate; hence, the link with fermentation quality was comparable to that of the bacterial community.

The quality of silage following anaerobic fermentation is intimately associated with the composition of the bacterial community, primarily due to the unfavorable anaerobic conditions that inhibit the reproduction of aerobic fungi and confer a significant survival advantage and dominance to anaerobic LAB, with the fermentation process also driven by bacterial metabolites, notably fatty acids (40). After 7 days of aerobic treatment, there was a negative correlation between the contents of LA and AA and each treatment. Each treatment was shown to be positively correlated with both the AN concentration and the pH. In the aerobic environment, the fungi in each treatment group became active and consumed LA and AA, resulting in an increase in the pH. Whereas the presence of oxygen results in a reduction in the inhibitory impact on *Clostridium*, oxygen increases the degradation of CP, and the AN content increases (38). The CCA results indicate that AVEO and LB may have a significant effect on increasing the levels of LA and AA, maintaining the CP content, and reducing the levels of AN, NDF, and ADF in triticale silage.

**Fig 5:**
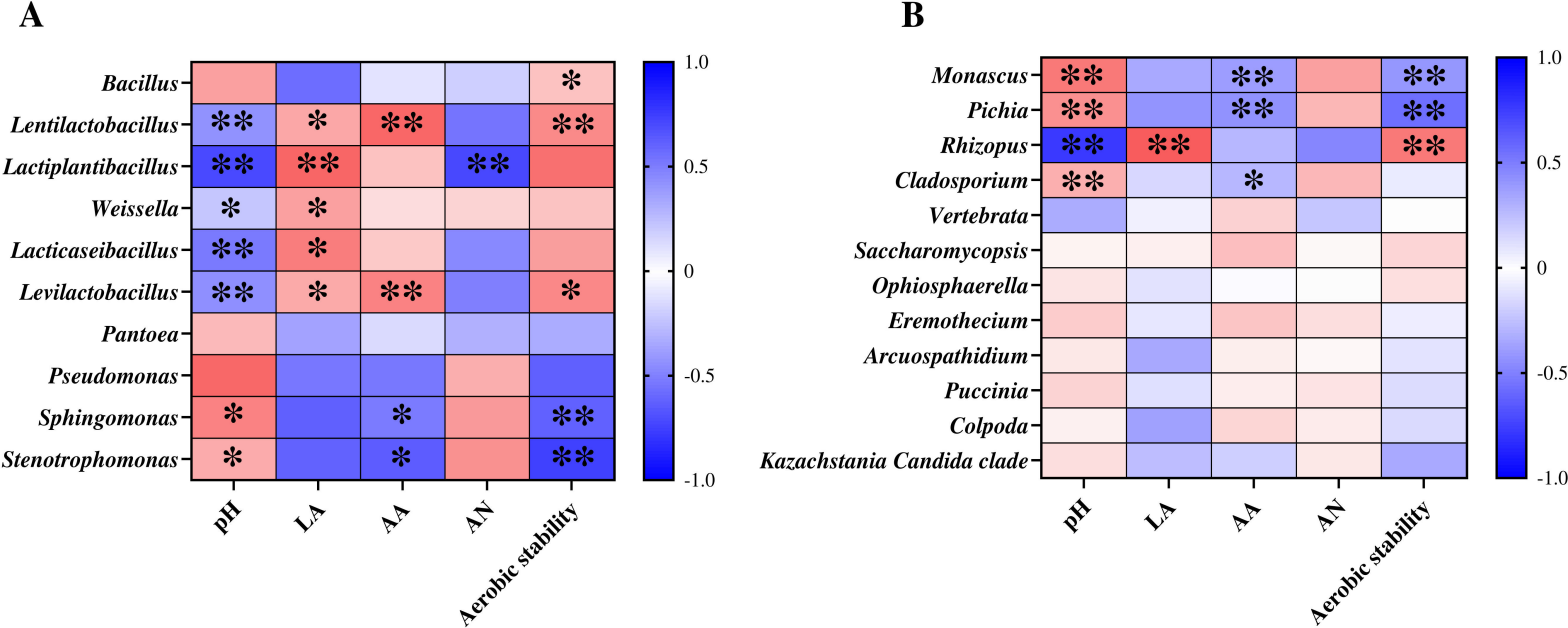
Spearman correlation heatmap between silage parameters and bacterial genera (**A**) and fungal genera (**B**) in triticale silage after 0 and 7 days of aerobic exposure. CK, control group (no exogenous inoculant); AVEO, *Amomum villosum* essential oil; LB, inoculation of *L. buchneri*; VA, vanillic acid. LA, lactic acid; AA, acetic acid; AN, ammonia nitrogen; *, *P* < 0.05; **, *P* < 0.01. Red squares represent positive correlations, and blue squares represent negative correlations.

To obtain a deeper understanding of the correlation between microbial dynamics and fermentation quality in triticale silage throughout aerobic exposure, genus-level Spearman correlation heatmaps of the primary bacteria and fungi were generated (Fig. 5). Within the bacterial community, there was a positive correlation between the *Bacillus* genus and aerobic stability (*P* < 0.05; Fig. 5A). This outcome corresponds to the results of Bai et al. (46), who proposed that *Bacillus* has a positive effect on improving the aerobic stability of silage. *Lactiplantibacillus*, *Weissella,* and *Lacticaseibacillus* were negatively correlated with the pH but positively correlated with LA (*P* < 0.05). This may be because all of these bacteria produce LA, causing the pH value to decrease. These observations are consistent with the findings presented in the studies by Ke et al. (53). However, *Lactiplantibacillus* was also negatively associated with the AN content. This is attributed to the fact that the presence of *Lactiplantibacillus* leads to an enhanced acidic environment, which can inhibit AN-producing bacteria (36). The *Lentilactobacillus* and *Levilactobacillus* genera were negatively correlated with the pH but positively correlated with the LA and AA contents and aerobic stability (*P* < 0.05). This was attributed to the fact that *Lentilactobacillus* and *Levilactobacillus* possess not only the ability to produce LA but also, as heterofermentative LAB, the ability to convert LA to AA (45), leading to an increase in aerobic stability. This discovery is consistent with the results published by Zhang et al. (12). *Sphingomonas* and *Stenotrophomonas* were negatively correlated with AA and aerobic stability but positively correlated with pH (*P* < 0.05). These findings are consistent with those of Wang et al. (54) and Liu et al. (44), who reported that *Sphingomonas* and *Stenotrophomonas* were positively correlated with the pH. The greater relative abundances of *Sphingomonas* and *Stenotrophomonas* in aerobic environments (Fig. 2B) suggested that these bacteria are associated with aerobic spoilage (42), which, coupled with the ability of AA to inhibit growth (23), is negatively correlated with AA and aerobic stability. Among the fungal communities, the genera *Monascus*, *Pichia,* and *Cladosporium* were negatively correlated with the AA content and positively correlated with the pH value (*P* < 0.05). The genera *Monascus* and *Pichia* were negatively correlated with aerobic stability (*P* < 0.05). This outcome is consistent with the previous results for fermentation indices and changes in the abundance of fungal genera (Table 2; Fig. 2D), where the relative abundances of *Monascus*, *Pichia,* and *Cladosporium* increased with increasing pH.

It has been shown that *Monascus* and *Pichia* are associated with aerobic spoilage (38, 44). This, together with the fact that AA has the capacity to inhibit growth, resulted in a negative correlation between AA and aerobic stability. Notably, there was a negative correlation between *Rhizopus* abundance and pH, and there was a positive correlation between *Rhizopus* abundance and LA and aerobic stability (*P* < 0.05). *Rhizopus* species can produce LA in an aerobic environment, which reduces the pH value (52), coupled with the fact that an acidic environment favors the aerobic stability of silage; therefore, *Rhizopus* species are positively correlated with LA and aerobic stability but negatively correlated with pH.

### Conclusion

Aerobic spoilage leads to nutrient losses in silage, leading to a significant reduction in storage efficiency and should be avoided in production practices. All of the additives tested in this work improved the aerobic stability of the triticale silage to varying degrees, with higher levels of LA, AA, and WSC and lower levels of NH_3_-N and NDF in the triticale silage with the additives throughout ensiling and subsequent exposure to air. AVEO enhanced the degradation of lignocellulose in triticale silage under aerobic conditions, providing a more fermentable substrate. Furthermore, the addition of AVEO maintained an acidic environment and reduced nutrient losses in the triticale silage, which was associated with an increased relative abundance of *Rhizopus* and decreased relative abundances of *Monascus* and *Sphingomonas*. Consequently, AVEO has the ability to positively impact the microbial community of triticale silage and thus improve fermentation quality and aerobic stability, which offers a scientific foundation for producing triticale silages of superior quality.

## Data Availability

The data sets used and/or analyzed during the current study are available from the corresponding author upon reasonable request.
